# A Width Measurement Method of Line Shape Based on Second Harmonic Peak and Modulation Amplitude

**DOI:** 10.3390/s23010476

**Published:** 2023-01-01

**Authors:** Shan Lin, Jun Chang, Jiachen Sun, Zihan Wang, Minghui Mao

**Affiliations:** 1 School of Information Science and Engineering and Shandong Provincial Key Laboratory of Laser Technology and Application, Shandong University, Qingdao 266200, China; 2 Key Laboratory of Laser and Infrared System of Ministry of Education, Shandong University, Qingdao 266200, China

**Keywords:** line width, second harmonic, modulation amplitude, wavelength modulation spectroscopy, absorption spectroscopy

## Abstract

The line width of different line shapes is a very important parameter in absorption spectroscopy sensing techniques. Based on the high sensitivity and low noise properties of wavelength modulation spectroscopy, we report a novel line width measurement method. After theoretically proving the relationship between line width, modulation amplitude and the amplitude of the second harmonic at the center frequency, the absorption lines of CH_4_ near 6046.96 cm^−1^ and CO_2_ 4989.97 cm^−1^ were chosen for simulation, and the relative errors of the line width between our method and theoretical data were kept at about 1%. A distributed feedback laser diode operating near 1653 nm with three different concentrations of CH_4_ was used for experimental validation, and the results were consistent with the numerical simulation. Additionally, since only the peaks of second harmonic need to be measured, the advantages of wavelength modulation can be utilized while reducing the difficulty of data acquisition.

## 1. Introduction

Tunable diode laser absorption spectroscopy (TDLAS) is widely used for trace gas detection because of its characteristics, such as non-contact measurement, high sensitivity, and fast response time [1,2,3,4]. As two main techniques of TDLAS, the variation of line shape has an important effect on both direct absorption spectroscopy (DAS) and wavelength modulation spectroscopy (WMS) [5,6,7,8,9]. Normally, the shapes can be described as Gaussian profiles, Lorentzian profiles and the convolution of these two profiles. The half width and half maximum (HWHM) of different profiles can be defined as the line width, which is an important factor that can determine the temperature, pressure, concentration and other characteristics. DAS can theoretically measure the line width, but in actual measurement, it is easily affected by factors such as light intensity fluctuation and baseline fitting accuracy, resulting in large errors. Compared with DAS, the interference of the background in WMS can be efficiently suppressed by high-frequency modulation, increasing the accuracy and sensitivity of the measurement [10,11]. However, in WMS, the line width is necessary to calculate the harmonics, and can not be directly measured. Normally, it is necessary to calibrate the gas concentration with the peak of second harmonic, and then determine the actual concentration from the calibration curve during the experiment. Therefore, the difference in calibration and experimental conditions leads to line width variation, which in turn leads to concentration errors. Although, relevant calibration-free methods have been proposed to simplify the calibration process which still require the line width as a known parameter for subsequent calculations [12,13,14]. Therefore, the measurement of line width is significant for TDLAS.

The importance of line width and line shape has attracted the attention of related researchers. Yang et al. reported absorption lines measurements of carbon disulfide near 4.6 μm, which would facilitate the development of calibrated carbon disulfide sensors [15]. Peng et al. recently presented a high-accuracy sinewave scanned DAS technique, in which a fitting routine based on the explicit baseline expression and accurate wavelength calibration was established [16]. By fitting the transmitted light in the time domain, the incident light intensity and absorption profile may be determined concurrently. To further simplify the measurement structure, Liang et al. proposed a novel line shape recovery method of DAS [17]. Based on the Fourier domain technique, they recovered the intensity scanning baseline and the absorption profile with just two filters. In addition, there are also many methods proposed for the line width in WMS. Stewart et al. proposed that when the modulation index *m* is less than 0.5, the first harmonic and the line shape can be approximately considered the same, and the measurement of the line shape can be realized by changing the phase between the reference and detection signal [18]. However, as the modulation coefficient increases, the accuracy of the measurement decreases rapidly. Lan et al. worded from the perspective of higher harmonics, and the ratios of the second and fourth harmonics at the line center have been used to measure line width [19]. Although there is a fixed point between the ratio of the second and fourth harmonics and the modulation index *m*, the signal-to-noise ratio of the fourth harmonic is poor and susceptible to background interference. Moreover, all these methods in WMS require different higher harmonics for calculation, and the process of data acquisition is relatively complicated.

Therefore, based on the above research, a novel method to measure the line width only using the amplitude of the second harmonic at the center frequency and the corresponding modulation amplitude is proposed in this work. We first theoretically obtained the relationship between the peak of second harmonic, the modulation amplitude and the HWHM of the line shape, and then evaluated the rationality of the proposed method through numerical simulation. Finally, to validate the precision, the data of CH_4_ near 6046.96 cm^−1^ with different concentrations were selected to compare the error measured by the method of this paper with database results using the experimental technique.

## 2. Theory

### 2.1. Basic Theory of WMS

In WMS, the signal with high angular frequency ω combined with a low-frequency scanning ramp is used to modulate the distributed feedback laser diode (DFB-LD), then the instantaneous laser output frequency ν(t) and output laser intensity $I_{0}\left(t\right)$ could, respectively be expressed as:

(1) $$\nu \left(t\right)=\nu_{c}+a\cos\left(\omega t+\psi \right)\;I_{0}\left(t\right)=I_{c}+\Delta I\cos\left(\omega t+\psi \right)$$

where ψ is the phase shift between intensity and frequency response, while a and ΔI represent the modulation amplitude of wavelength and intensity around $\nu_{c}$ and $I_{c}$, which are the slowly varying values of the average wavelength and intensity.

According to the Beer–Lambert relation, the transmitted intensity $I_{t}\left(\nu \right)$ is described by:

(2) $$I_{t}\left(\nu \right)=I_{0}\left(\nu \right)\left[1-\alpha \left(\nu \right)L\right]$$

where $I_{0}\left(\nu \right)$ is the incident intensity, and L is the optical absorbing path. α(ν) is the absorption coefficient at frequency ν, which is a period-even function and can be expanded in a Fourier cosine series:

(3) $$\alpha \left(\nu \right)=\alpha \left[\nu_{c}+a\cos\left(\omega t+\psi \right)\right]=\sum_{n=0}^{\infty }H_{n}\left(\nu_{c}\right)\cos n\omega t$$

where the Fourier components $H_{n}\left(\nu_{c}\right)$ can be expressed as:

(4) $$H_{n}\left(\nu_{c}\right)=\frac{2^{1-n}}{n!}a^{n}{\frac{d^{n}\alpha \left(\nu \right)}{d\nu^{n}}|}_{\nu =\nu_{c}},n\geq 1$$

Considering the case in which the pressure is sufficient to ensure that pressure broadening dominates, α(ν) could be described by a Lorentz profile:

(5) $$\alpha \left(\nu \right)=\frac{1}{1+{\left[\frac{\nu \left(t\right)-\nu_{0}}{\gamma }\right]}^{2}}$$

where γ is the half width at half maximum (HWHM) and $\nu_{0}$ is the center of the absorption line.

### 2.2. The Theory of the Proposed Method

Following the work of Arndt [20], we define a dimensionless parameter $x=\frac{\nu_{c}-\nu_{0}}{\gamma }$; the result for the second Fourier coefficient is:

(6) $$H_{2}\left(x\right)=\frac{4\gamma^{2}}{a^{2}}-\frac{\sqrt{2}\gamma^{2}}{a^{2}}\frac{\left(M+1-x^{2}\right)\sqrt{\left[\sqrt{\left(M^{2}+4x^{2}\right)}+M\right]}+4x\sqrt{\left[\sqrt{\left(M^{2}+4x^{2}\right)}-M\right]}}{\sqrt{\left(M^{2}+4x^{2}\right)}}$$

where $M=1-x^{2}+\frac{a^{2}}{\gamma^{2}}$.

Then, the maximum value of the second harmonic (2*f*) signal at center frequency can be expressed as:

(7) $$H_{2}\left(0\right)=I_{0}\alpha_{0}CL\left[-4{\left(\frac{\gamma }{a}\right)}^{2}+\sqrt{2}{\left(\frac{\gamma }{a}\right)}^{2}\frac{\left[2+{\left(\frac{a}{\gamma }\right)}^{2}\right]\sqrt{2\left[1+{\left(\frac{a}{\gamma }\right)}^{2}\right]}}{1+{\left(\frac{a}{\gamma }\right)}^{2}}\right]$$

To establish the relationship between modulation amplitude a and line width γ, we define R as:

(8) $$R=\frac{H_{2-1}\left(0\right)}{H_{2-2}\left(0\right)}={\left(\frac{a_{2}}{a_{1}}\right)}^{2}\frac{\sqrt{1+{\left(\frac{a_{1}}{\gamma }\right)}^{2}}+1/\sqrt{1+{\left(\frac{a_{1}}{\gamma }\right)}^{2}}-2}{\sqrt{1+{\left(\frac{a_{2}}{\gamma }\right)}^{2}}+1/\sqrt{1+{\left(\frac{a_{2}}{\gamma }\right)}^{2}}-2}$$

By detecting a set of the peak values of 2*f* signals and the corresponding modulation amplitude, the linewidth γ can be calculated in real-time.

## 3. Simulation

**Figure 1:**
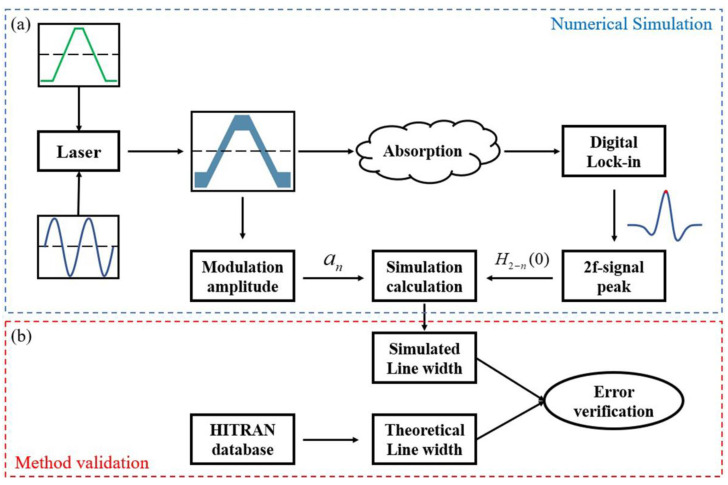
Simulation process. (**a**) Numerical simulation of harmonic signal; (**b**) Simulation validation of the proposed method.

**Figure 2:**
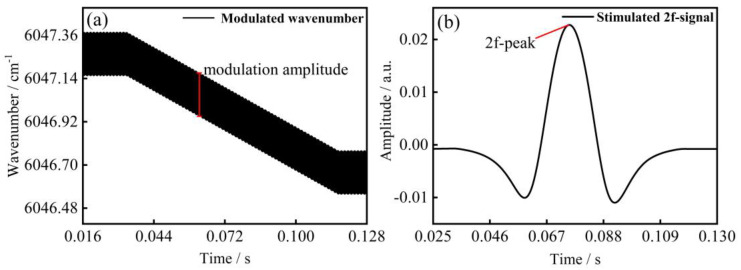
Numerical simulation results. (**a**) Modulation amplitude; (**b**) 2f-signal.

To verify the precision and reliability of the method proposed in this paper, we conducted a simulation using MatLab, and the whole process can be divided into numerical simulation and method validation, as shown in Figure 1. The DFB-LD is driven by a low-frequency scanning ramp, and then the modulation is superimposed with a high-frequency sinusoidal signal in kHz. By changing the voltage amplitude of the modulated signal, different instantaneous optical frequency outputs can be simulated. According to Equation (1), modulation amplitude a reflects the amplitude change of the modulated signal, as shown in Figure 2a.

Then, as shown in Figure 2b, the peak value at the center frequency is extracted from the second harmonic signal obtained by digital lock-in, and the line width under different conditions is calculated in combination with the corresponding modulation amplitude obtained before, according to Equation (8). The accuracy of the proposed method is verified by comparing the simulated results with the theoretical line widths in the database.

The CH_4_ and CO_2_ were chosen as the simulation objects, and the absorption lines of them near 6046.96 cm^−1^ and 4989.97 cm^−1^ were selected to recover the line widths, respectively. The spectroscopic parameters of these transitions are shown in Table 1 and Table 2 (all data are taken from HITRAN database [21,22]).

**Figure 3:**
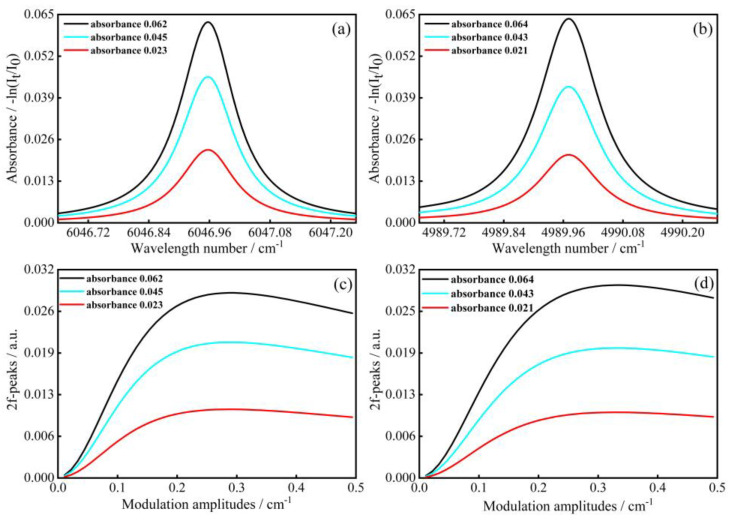
Different absorbance of (**a**) CH_4_ and (**b**) CO_2_. 2f-peaks corresponding to different modulation amplitudes of (**c**) CH_4_ and (**d**) CO_2_.

To obtain the different second harmonic peaks and modulation amplitudes, the parameters for these two simulation objects need to be determined first. Considering the approximation of absorbance is less than about 0.1 in low absorption level, and making the simulation closer to the real measurement, the temperature, pressure and absorption path of the two objects were the same, with 298 K, 1 atm, 300 cm, respectively. As shown in Figure 3a,b, considering the variation of absorbance, three different ranges around 0.02, 0.04 and 0.06 were taken for CH_4_ and CO_2_, respectively. According to the above conditions, we calculated the HWHM ratio of Lorentzian and Gaussian line shapes for each of the two objects at different concentrations, and all were much greater than five. Therefore, the Lorentzian line shape was appropriate to use for fitting in this paper. Then, by changing the amplitude of the modulation voltage, multiple different sets of modulation amplitudes and their correlating second harmonic amplitudes were obtained, as shown in Figure 3c,d.

**Figure 4:**
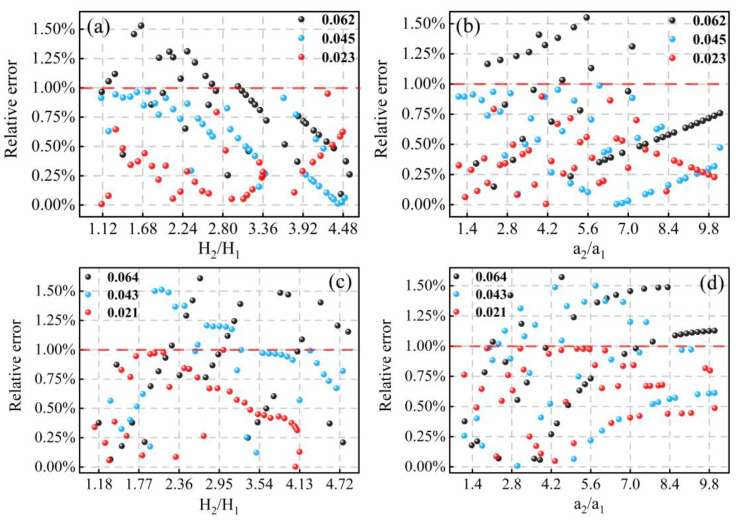
Relative errors of line width for different absorbance of (**a**,**b**) CH_4_ and (**c**,**d**) CO_2_.

The different modulation amplitudes a and their corresponding second harmonic amplitudes at center frequency were randomly combined from the simulation results of each sample. Then, according to Equation (8), the ratios of modulation amplitudes and the ratios of second harmonic amplitudes were calculated to verify the accuracy of the method proposed in this paper. The errors between the theoretical line widths of CH_4_ and CO_2_ and the results calculated by the method in this paper are shown in Figure 4. It can be seen from the simulation results that the errors are consistently less than 1% from the simulation results. The overall error stayed in a low range; especially at low absorbance, the error was less than 1%. As the absorbance approaches or even exceeds 0.1, the amplitude of the second harmonic gradually deviates from the linear relationship with the concentration, making the error in the simulated linewidth and the result calculated by the method gradually increase.

## 4. Experiment

**Figure 5:**
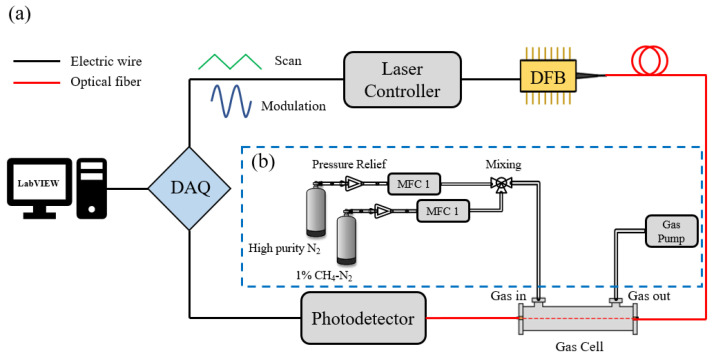
(**a**) Diagram of the experiment scheme; (**b**) Diagram of gas mixing system.

The diagram of the experiment scheme is shown in Figure 5. The R(3) line near 6046.9636 cm^−1^ of CH_4_ was applied in the detection with temperature T = 296 K, pressure P = 1 atm and L = 300 cm. Therefore, the fiber-coupled DFB-LD (SWLD-165310S22-01, Allwave Devices Inc., Xi’an, China), operated at 1653 nm, and was used as the spectroscopic source which was controlled by the laser diode controller (LDC501, Stanford Research Systems Inc., Sunnyvale, CA, USA). The modulated signal superimposed by a 4 Hz triangle wave and 6 kHz sine wave was output by the data acquisition card (USB-6212, NI). The output of the DFB-LD was propagated to a fiber-coupled Herriott cell (300 cm path length) and then converted to voltage signal by the photodetector module (PDB450C, Thorlabs, Shanghai, China). The harmonic signal was extracted by the digital lock-in amplifier with a reference signal generated by the data acquisition card. Note that the signal generator, digital lock-in amplifier, and data processing were all integrated with a LabVIEW-based program to simplify the system.

**Figure 6:**
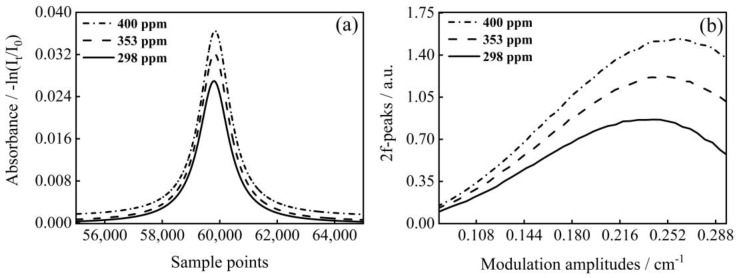
(**a**) Absorbance of different concentrations of CH_4_; (**b**) Experimental measured 2f-peaks and corresponding modulation amplitudes.

**Figure 7:**
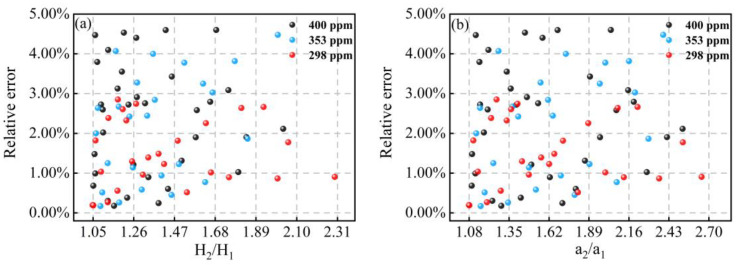
Relative errors of line width for different concentrations of CH_4_ with different 2f-peaks ratios (**a**) and modulation amplitude ratios (**b**).

The gas mixing system for the preparation of methane concentration is illustrated in Figure 5b. The standard gas of 1% CH_4_/N_2_ was diluted with high-purity N_2_. Two mass flow controllers (CS200A, Beijing Sevenstar Flow Co., Ltd., Beijing, China) with different flow rates were employed to control the flow of the two gas paths to achieve the preparation of different concentrations of CH_4_. As shown in Figure 6, we mixed three different concentrations of CH_4_ and measured the transmission signals and the 2f-peaks corresponding to different modulation amplitudes at each concentration. Because of the limitation of the tuning range of our DFB-LD, there was a small range of variation in the experimentally measured modulation amplitude. Then, the line widths under experimental conditions were calculated based on the transmission, and were compared with the results of the method proposed in this paper and the errors are shown in Figure 7. It can be seen that the experimental results were consistent with the simulated data as a whole, and the calculation results were more accurate at low concentrations. However, the overall line width error calculated according to the experimental data is slightly larger than the theoretical simulation, which was greatly affected by the accuracy of the laser modulation amplitude.

## 5. Conclusions

In this paper, we propose a novel WMS-based method for measuring the width of line shape. Since the second harmonic amplitude is a function of modulation amplitude and line width, we consider that the line width can be calculated from the amplitudes at the center frequency of the second harmonic, corresponding to multiple sets of different modulation amplitudes. Therefore, based on harmonic theories, we first theoretically deduced the new method proposed in this paper. Then, to demonstrate the reliability and accuracy of this method, the absorption lines near 6046.96 cm^−1^ and 4989.97 cm^−1^ were chosen for numerical simulations and experiments, the results of which showed that the method proposed in this paper can measure the width of line shape well in different conditions. It is worth mentioning that this paper presents more of a new way of thinking about line width measurements. Compared with using DAS to measure line width, our second harmonic-based method takes advantage of the high signal-to-noise ratio of WMS, while avoiding the use of higher harmonic signals that are susceptible to background interference. We believe that this method can be applied to different experimental conditions, and the higher the measurement accuracy of the modulation amplitude and the second harmonic amplitude, the better the results of this method.

## Figures and Tables

**Table 1:** Spectroscopic parameters for the selected CH_4_ transition near 6046.96 cm^−1^ (298 K).

Wavenumber (cm^−1^)	S (cm^−2^ atm^−1^)	γ_air_ (cm^−1^ atm^−1^)	γ_self_ (cm^−1^ atm^−1^)	E″ (cm^−1^)
6046.943	0.0192	0.0648	0.079	62.8758
6046.952	0.0227	0.077	0.079	62.8768
6046.964	0.0355	0.0575	0.079	62.8782

**Table 2:** Spectroscopic parameters for the selected CO_2_ transition near 4989.97 cm^−1^ (298 K).

Wavenumber (cm^−1^)	S (cm^−2^ atm^−1^)	γ_air_ (cm^−1^ atm^−1^)	γ_self_ (cm^−1^ atm^−1^)	E″ (cm^−1^)
4989.952	1.11 × 10^−8^	0.0602	0.066	3222.251
4989.971	0.0326	0.074	0.1	106.1297
4990.004	1.85 × 10^−8^	0.0697	0.09	1653.074

## Data Availability

The data presented in this study are available on request from the corresponding author.
